# Glutamatergic lateral habenula neurons modulate consolidation of associative memories

**DOI:** 10.3389/fnbeh.2025.1646689

**Published:** 2025-07-29

**Authors:** Snigdha Srivastava, I-Ching Wang, Mikhail Y. Kochukov, Jessica L. Swanson, Mauro Costa-Mattioli, Benjamin R. Arenkiel

**Affiliations:** ^1^Genetics and Genomics Graduate Program, Baylor College of Medicine, Houston, TX, United States; ^2^Department of Molecular and Human Genetics, Baylor College of Medicine, Houston, TX, United States; ^3^Jan and Dan Duncan Neurological Research Institute, Texas Children’s Hospital, Houston, TX, United States; ^4^Medical Scientist Training Program, Baylor College of Medicine, Houston, TX, United States; ^5^Department of Neuroscience, Baylor College of Medicine, Houston, TX, United States; ^6^Department of Neuroscience, University of Minnesota Medical School, Minneapolis, MN, United States; ^7^Altos Labs, Inc., Bay Area Institute, Redwood City, CA, United States

**Keywords:** lateral habenula, ketamine, memory, glutamatergic, chemogenetics

## Abstract

**Introduction:**

Despite the rise in psychiatric disorders worldwide, the underlying brain circuits responsible for these devastating conditions remain elusive. The lateral habenula (LHb) has emerged as a key brain structure in depression studies due to its hyperactive state in both patients and animal models. While this aligns with known roles in driving aversive states and regulating serotonin release, it is still unclear how acute and transient activity changes in the LHb can influence higher order cognitive processes such as learning, memory, and behavioral adaptation. Given the importance of these processes to psychiatric conditions, understanding how LHb activity impacts cognitive function allows novel insights into the neurobiological mechanisms of disorders like depression.

**Methods:**

Towards this goal, we used chemogenetic activation to temporarily excite glutamatergic neurons in the mouse LHb and assessed impacts on associative memory.

**Results and Discussion:**

Surprisingly, we found that transient activation of LHb impaired long-term memory, without affecting anxiety or depression-like behaviors. Specifically, post-training activation of LHb glutamatergic neurons disrupted object recognition and reward-based associative long-term memory, while sparing fear associated long-term memory. The memory impairment was restricted to a critical temporal window post-training/conditioning that corresponded with the consolidation stage of long-term memory. Strikingly, pairing LHb glutamatergic neuronal activation with systemic ketamine administration rescued the long-term memory deficits, indicating that LHb glutamatergic neurons modulate consolidation of associative memories via a NMDA-mediated mechanism. Together, these findings support a novel role for LHb glutamatergic neuronal activity in the consolidation of associative long-term memories.

## Introduction

Evolutionarily conserved brain circuits that govern reward and aversion are essential to animal survival. These circuits allow animals to sense the external world and update their internal state to drive appetitive or avoidance behaviors, while relaying salience and valence information to higher order cognitive centers (Hu, 2016). At their core, these evolutionarily conserved circuits ensure access to satisfying goal directed behaviors such as feeding and social interactions, while also ensuring survival (Xu et al., 2019; Karamihalev and Gogolla, 2022). Reward and aversion circuits in the brain are primarily found within the limbic system, an interconnected network of brain networks that give rise to complex behaviors, cognition, and emotions. Furthermore, limbic structures are well established to play roles in mood regulation, and thus, represent a major focal point for investigations into mood stability and neuropsychiatric illnesses (Hu, 2016).

One of the key regions within the limbic system is the habenula, a highly conserved midbrain structure that is divided into two subnuclei, the medial habenula (MHb) and lateral habenula (LHb) in mammals, or dorsal and ventral habenula in fish and amphibians. The LHb, sometimes referred to as the aversion or “antireward” center of the brain, has received a significant amount of interest due to its unique position within the limbic system (Hu et al., 2020; Matsumoto and Hikosaka, 2007, 2009). The LHb receives input from diverse limbic structures in the forebrain and basal ganglia and sends projections out to modulate major mood regulation centers, including the serotonergic and dopaminergic systems (Hu et al., 2020). Thus, the LHb plays a critical role in reward/aversion processing, as well as emotional regulation through its neuromodulatory targets (Groos and Helmchen, 2024; Flerlage et al., 2024; Metzger et al., 2017; Zhou et al., 2019). Previous studies of this region in macaques describe a significant increase in habenular activity when subjects did not receive an expected reward (Matsumoto and Hikosaka, 2007, 2009). This negative reward prediction error became a hallmark for understanding LHb responses to external stimulus information, and highlighted this structure as a key effector of aversive behavior.

The LHb is primarily comprised of excitatory glutamatergic cells and a relatively small population of inhibitory GABAergic cells (Hu, 2016). It is known to be activated by diverse aversive stimuli, including electric foot shocks and predator cues, and is ultimately thought to drive aversive behaviors, including escape and freezing responses in mice (Lecca et al., 2017; Yang et al., 2016; Congiu et al., 2023). When artificially activated using optogenetic approaches, the LHb has been shown to drive robust avoidance and aversion through glutamatergic excitation in this region (Hu et al., 2020). Interestingly, in rodent and zebrafish models of depression, the LHb has been highlighted as the major region of hyperactivity associated with depression (Yang et al., 2018b; Andalman et al., 2019). This correlates with recent evidence of habenular hyperactivity linked to depression in fMRI imaging studies of human patients (Yang et al., 2018b; Gosnell et al., 2019a; Li et al., 2013). Together, these studies have provided mounting evidence for a link between the LHb and depression, and recent work in this field has found that neurons within the LHb show a specific kind of burst firing hyperactivity that can serve as a potential biomarker for depressive states. In fact, LHb burst firing has been shown to drive depression by artificial stimulation experiments in rodents, and this burst firing is considered a major target of ketamine antidepressant therapy due to ketamine’s action in reducing burst firing in hyperactive LHb neurons (Yang et al., 2018a).

One notable aspect of LHb physiology is its position at the interface between emotion and higher order cognitive processes like learning and memory, especially given the high prevalence of cognitive impairment in patients with depression (Rock et al., 2014; Mendelsohn et al., 2009; Culpepper et al., 2017). Over the past decade, recent work has begun to shed light on how the LHb acts in concert with other brain regions like the amygdala, hippocampus, and medial prefrontal cortex to mediate learning and memory (Mathis and Lecourtier, 2017). LHb activity has been shown to be critical for online processing of specific types of cued memories (Tronel and Sara, 2002) and LHb activity increases with exposure to contextual fear memory cues (González-Pardo et al., 2012). Moreover, several studies have implicated a role of LHb in the acquisition or retention of aversive memory (Tomaiuolo et al., 2014; Ilango et al., 2013; Shumake et al., 2010; Stamatakis and Stuber, 2012). Pharmacological inhibition of LHb activity has also been shown to impair specific aspects of spatial memory processing (Mathis and Lecourtier, 2017; Mathis et al., 2015), while optogenetic stimulation of excitatory forebrain inputs to the LHb has been shown to drive reflexive aversive states that block memory (Swanson et al., 2022). These studies suggest that the LHb serves as a potential gateway between emotion and cognition, allowing for the integration of experience and emotion to guide memory-based behavioral adaptation.

While such studies provide a critical foundation that identifies the LHb in driving naturalistic aversion, memory, and the pathophysiology of depression, there is still a significant dearth of information regarding the cell type-specific and/or neural circuit contributions within this brain region that underlie its role in learning and memory. In this study, we sought to better understand how LHb activity impacts complex behavioral memory by targeting glutamatergic neurons within the LHb for chemogenetic hyperactivation. We hypothesized that this manipulation would shed light on the mechanisms of how memory is impacted in a depressive state. However, we discovered a novel depression-independent role of LHb glutamatergic neuron activity in the consolidation of associative memories.

## Materials and methods

### Animal care

All procedures performed on mice were carried out in accordance with the ethical guidelines of the National Institutes of Health and approved by the institutional review board (IACUC Baylor College of Medicine) under protocol #AN-5596. All experiments were conducted using *Vglut2-Cre* adult mice (<6 month age) purchased from Jackson Laboratories (Slc17a6^tm2 (cre)Lowl^/J Stock No. 016963) (Vong et al., 2011). Genotyping for vGlut2-Cre was done using the following primers from Jackson Laboratories: Mutant Reverse “ACA CCG GCC TTA TTC CAA G” (Primer 13007), Common “AAG AAG GTG CGC AAG ACG” (Primer 32667), and Wild type Reverse “CTG CCA CAG ATT GCA CTT GA” (Primer 32668). To obtain heterozygotes (vGlut2-Cre^+/−^), vGlut2-Cre homozygous mice (vGlut2-Cre^+/+^) were crossed to C57BL/6NJ wildtype mice (Jackson labs Stock No. 005304). All mice were housed with food and water available ad libitum in a 12-h light/dark environment. Both male and female mice were used for all experiments, and mice were randomly allocated to experimental groups. For *ex vivo* and *in vivo* experiments, adult mice aged >8 weeks were used, unless otherwise described.

### Experimental design

**Figure 1:**
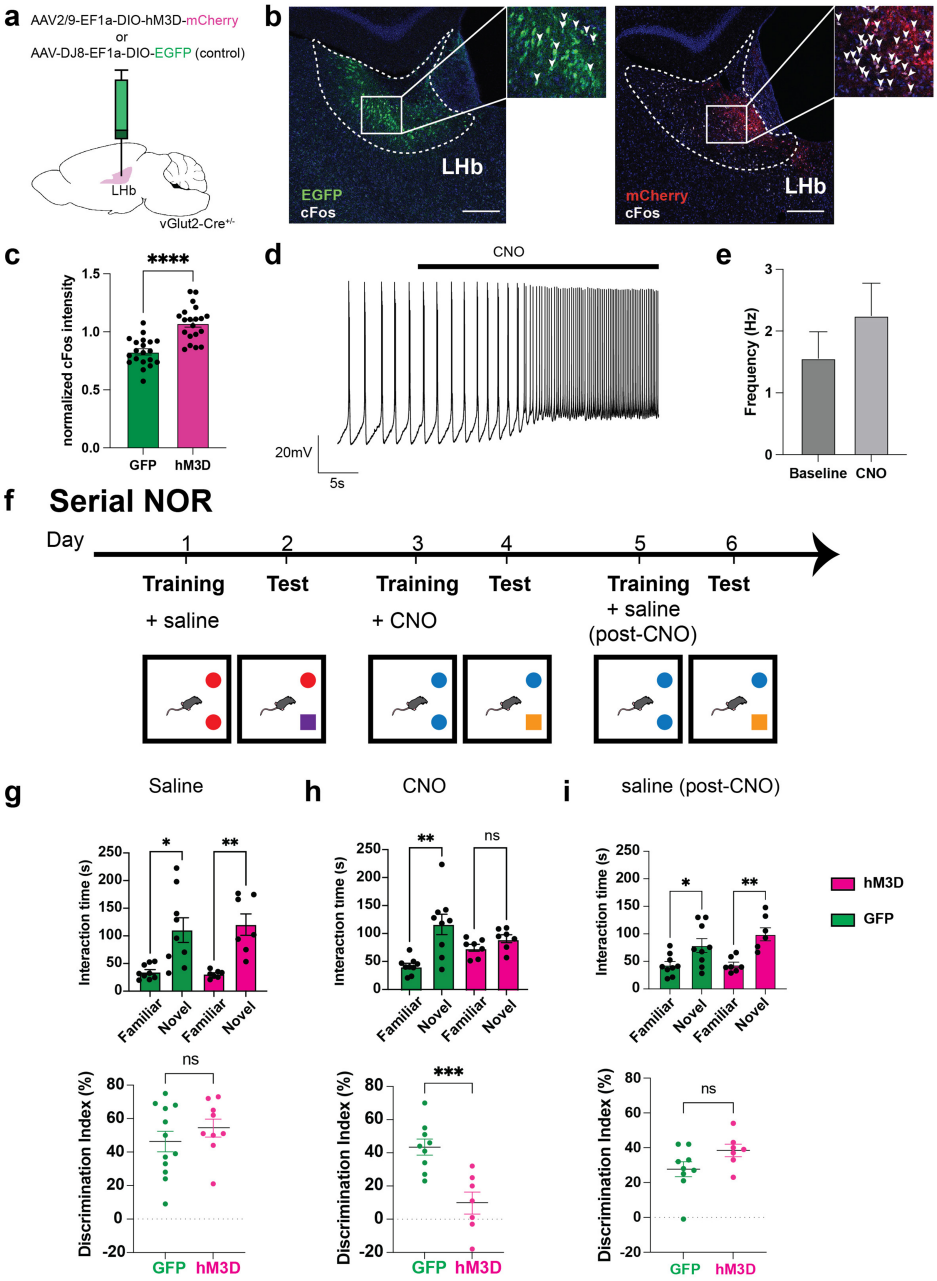
Chemogenetic activation of glutamatergic LHb neurons disrupts memory. **(a)** Experimental setup for chemogenetic activation experiments, in which *vGlut2-Cre* mice are used for stereotaxic LHb injections of AAV-Ef1a-DIO-hM3D-mCherry (chemogenetic actuator) or AAV-Ef1a-DIO-EGFP (control) viruses. **(b)** Representative image of viral expression of EGFP (left) and mCherry-tagged hM3D (right) in LHb at Bregma −1.5 and Bregma −1.45 respectively, with co-stain for cFos activity-dependent marker expression in mice injected with CNO prior to perfusion and tissue dissection. Arrowheads mark cFos+ cells. Scale bar = 200 μm. **(c)** Quantification of DAPI-normalized cFos intensity in GFP and hM3D-injected mice (*n* = 3 mice per group, 3–4 sections from anterior to posterior surveyed per animal). To calculate these data, average cFos intensity was normalized to DAPI average intensity in user-defined LHb ROI. Statistical significance evaluated using unpaired Student’s *t*-test, error bars represent SEM (*p* < 0.0001, alpha = 0.05). **(d)** Representative *ex vivo* electrophysiological trace from hM3D-expressing cell in the LHb with bath application of CNO. Recordings were taken from 8 cells across 6 mice. **(e)** Average firing frequency of hM3D-expressing cells before and after CNO bath application in 18 cells and 14 cells, respectively. Statistical significance evaluated using unpaired Student’s *t*-test, error bars represent SEM (*p* = 0.3138, alpha = 0.05). **(f)** Behavioral paradigm for serial novel object recognition tasks. In this version, mice are trained on two identical objects on day 1, then injected with either saline or CNO. 24 h later, they are assessed on how well they can discriminate between familiar and novel object. **(g)** Time spent with familiar and novel objects (top) was measured in both GFP control (shown in green, *n* = 12) and hM3D experimental (shown in magenta, *n* = 9) groups when injected with saline post-training. Proper memory function is indicated by significantly higher time spent with novel object compared to familiar object. Statistical significance between novel and familiar object investigation time calculated using One-way ANOVA with Sidak multiple comparisons (GFP familiar vs. novel adjusted *p* = 0.0002, hM3D familiar vs. novel adjusted *p* = 0.0002, alpha = 0.05). Discrimination index (bottom) calculated as [((time investigating novel object - time investigating familiar object)/total investigation time) * 100] on test day of novel object recognition. A discrimination index over 0 indicates a preference for investigating a novel object, as expected. Statistical significance between DI hM3D and DI GFP calculated using unpaired Student’s *t*-test (*p* = 0.3558, alpha = 0.05). **(h)** Time spent with familiar and novel objects (top) measured in both GFP control (shown in green, *n* = 9) and hM3D experimental (shown in magenta, *n* = 7) groups injected with CNO post-training. Statistical significance between novel and familiar object investigation time calculated by One-way ANOVA with Sidak multiple comparisons (GFP familiar vs. novel adjusted *p* < 0.0001, hM3D familiar vs. novel adjusted *p* = 0.5917, alpha = 0.05). Discrimination index (bottom) on test day of novel object recognition. Statistical significance between DI hM3D and DI GFP calculated by unpaired Student’s *t*-test (*p* = 0.0009, alpha = 0.05). **(i)** Time spent with familiar and novel objects (top) measured in both GFP control (shown in green, *n* = 9) and hM3D experimental (shown in magenta, *n* = 7) groups injected with saline post-training after CNO washout. Statistical significance between novel and familiar object investigation time calculated by Kruskal-Wallis test with multiple comparisons (GFP familiar vs. novel *p* = 0.0444, hM3D familiar vs. novel adjusted *p* = 0.0019, alpha = 0.05). Discrimination index (bottom) on test day of novel object recognition. Statistical significance between DI hM3D and DI GFP calculated by Mann–Whitney test (*p* = 0.0961, alpha = 0.05).

**Figure 2:**
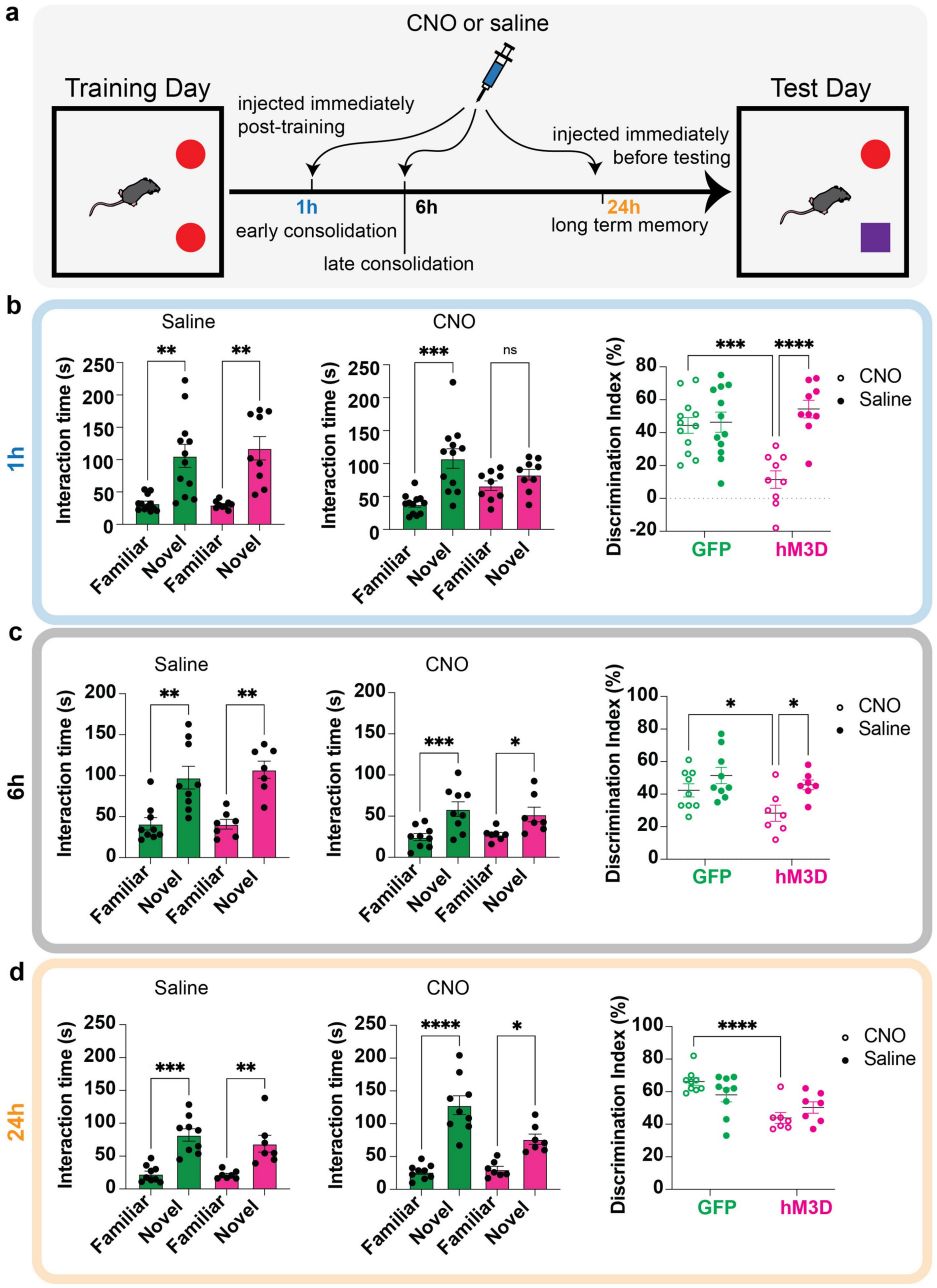
Temporal profile of memory vulnerability to glutamatergic LHb neuronal activation. **(a)** Behavioral paradigm for temporal profiling of memory vulnerability using novel object recognition tasks. Mice are trained on two identical objects on day 1, then injected with either saline or CNO to chemogenetically excite vGlut2+ LHb cells at distinct time points (immediately post-training to test early consolidation, 6 h post-training to test late consolidation, and 24 h post-training to test long term memory storage). The next day, they are assessed on how well they can discriminate between familiar and novel object. **(b)** Time spent with familiar and novel objects measured in GFP control (green bars, *n* = 12) and hM3D experimental (magenta bars, *n* = 9) groups injected with saline (left) or CNO (middle) immediately post-training. Statistical significance between novel and familiar object investigation time calculated by One-way ANOVA with Sidak multiple comparisons. *Saline:* GFP familiar vs. novel adjusted *p* = 0.0020, hM3D familiar vs. novel adjusted *p* = 0.0019, alpha = 0.05. *CNO*: GFP familiar vs. novel adjusted *p* = 0.0006, hM3D familiar vs. novel adjusted *p* = 0.1275, alpha = 0.05. Discrimination index (calculated as in Figure 1) for GFP and hM3D mice when injected with saline or CNO immediately post-training (right). Statistical significance between discrimination indices of GFP and hM3D mice with CNO and saline treatments calculated by 2-way ANOVA with Sidak multiple comparisons. GFP (CNO vs. saline): *p* = 0.7940, alpha = 0.05. hM3D (CNO vs. saline): *p* < 0.0001, alpha = 0.05. CNO (GFP vs. hM3D): *p* = 0.0002, alpha = 0.05. Saline (GFP vs. hM3D): *p* = 0.3160, alpha = 0.05. **(c)** Time spent with familiar and novel objects measured in GFP control (green bars, *n* = 9) and hM3D experimental (pink bars, *n* = 7) groups injected with saline (left) or CNO (middle) 6 h post-training. Statistical significance between novel and familiar object investigation time calculated by One-Way ANOVA with Sidak multiple comparisons and Kruskal–Wallis test with multiple comparisons for parametric and non-parametric data, respectively. *Saline*: GFP familiar vs. novel *p* = 0.0024, hM3D familiar vs. novel *p* = 0.0023, alpha = 0.05. *CNO:* GFP familiar vs. novel adjusted *p* = 0.0008, hM3D familiar vs. novel adjusted *p* = 0.0302, alpha = 0.05. Discrimination index for GFP and hM3D mice when injected with saline or CNO 6 h post-training (right). Statistical significance between discrimination indices of GFP and hM3D mice with CNO and saline treatments calculated by mixed effects model with multiple comparisons. GFP (CNO vs. saline): *p* = 0.1337, alpha = 0.05. hM3D (CNO vs. saline): *p* = 0.0145, alpha = 0.05. CNO (GFP vs. hM3D): *p* = 0.0341, alpha = 0.05. Saline (GFP vs. hM3D): *p* = 0.3713, alpha = 0.05. **(d)** Time spent with familiar and novel objects measured in GFP control (green bars, *n* = 9) and hM3D experimental (pink bars, *n* = 7) groups injected with saline (left) or CNO (middle) 24 h post-training. Statistical significance between novel and familiar object investigation time calculated by One-Way ANOVA with Sidak multiple comparisons and Kruskal–Wallis test with multiple comparisons for parametric and non-parametric data, respectively. *Saline:* GFP familiar vs. novel adjusted *p* = 0.0001, hM3D familiar vs. novel adjusted *p* = 0.0057, alpha = 0.05. *CNO*: GFP familiar vs. novel adjusted *p* < 0.0001, hM3D familiar vs. novel adjusted *p* = 0.0180, alpha = 0.05. Discrimination index for GFP and hM3D mice when injected with saline or CNO 24 h post-training (right). Statistical significance between discrimination indices of GFP and hM3D mice with CNO and saline treatments calculated by mixed effects model with multiple comparisons. GFP (CNO vs. saline): *p* = 0.0821, alpha = 0.05. hM3D (CNO vs. saline): *p* = 0.2241, alpha = 0.05. CNO (GFP vs. hM3D): *p* < 0.0001, alpha = 0.05. Saline (GFP vs. hM3D): *p* = 0.1248, alpha = 0.05.

**Figure 3:**
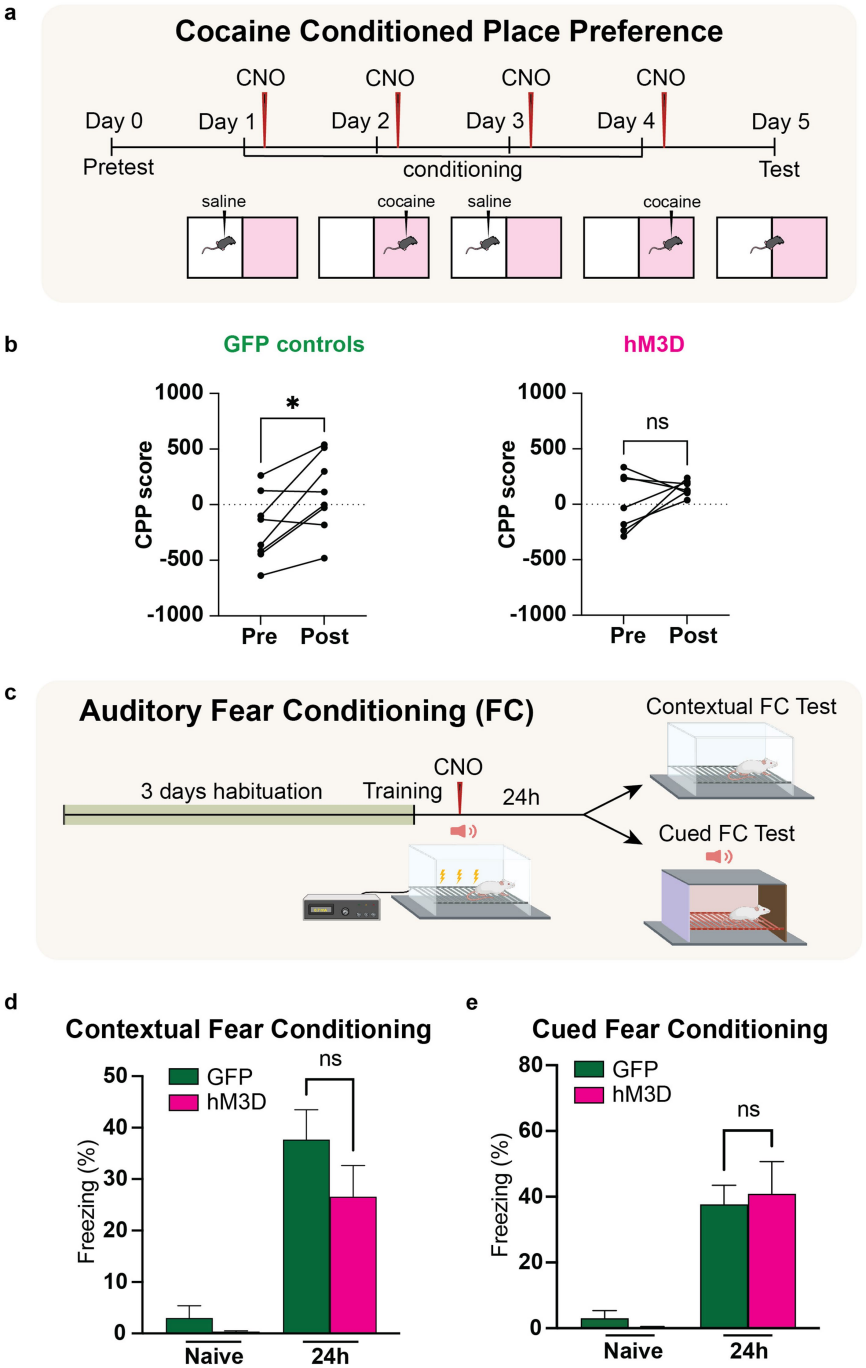
Chemogenetic activation of LHb glutamatergic neurons disrupts reward memory but leaves fear memory intact. **(a)** Experimental paradigm for cocaine conditioned place preference with chemogenetic activation of vGlut2+ LHb neurons. CNO is injected post-training for each day of conditioning to activate LHb glutamatergic neurons. **(b)** Change in preference in GFP control animals (*n* = 6) pre- and post-cocaine CPP (left). CPP score calculated as [time spent in cocaine chamber − time spent in saline chamber]. Statistical significance between pre- and post-conditioning CPP score for GFP mice calculated by paired Student’s *t*-test (*p* = 0.0260, alpha = 0.05). Change in preference in hM3D experimental animals (*n* = 7) pre- and post-cocaine CPP (right). Statistical significance between pre- and post-conditioning CPP score for hM3D mice calculated by paired Student’s *t*-test (*p* = 0.2447, alpha = 0.05). **(c)** Experimental paradigm for auditory fear conditioning with chemogenetic activation of vGlut2+ LHb neurons (see methods and materials for full description). CNO is injected post-training to activate LHb glutamatergic neurons. **(d)** Contextual fear conditioning test. Freezing response of GFP control (*n* = 6) and hM3D experimental (*n* = 7) groups to conditioned context before and after training. Statistical significance determined by Mann–Whitney test: GFP vs. hM3D freezing % (*p* = 0.4452, alpha = 0.05). **(e)** Auditory fear conditioning test. Freezing response of GFP control (*n* = 6) and hM3D experimental (*n* = 7) groups to conditioned auditory tone before and after training. Statistical significance determined by Mann–Whitney test: GFP vs. hM3D freezing % (*p* = 0.8357, alpha = 0.05).

A cohort of animals were first tested for serial novel object recognition tasks with CNO-mediated chemogenetic manipulation immediately after training days (see Figure 1). This same cohort was then tested serially with CNO injections at different time points after training to determine stage-specificity of memory vulnerability to chemogenetic manipulation (see Figure 2). Of this initial cohort, a subset of these animals was advanced to cocaine CPP, followed by fear conditioning tests (see Figure 3), after which they were retired from behavioral testing. All other experiments were performed separately with distinct cohorts of animals.

### Stereotaxic viral injections

For all stereotaxic surgeries, mice were anesthetized using ~1–3% vaporized isoflurane with oxygen. A stereotaxic instrument connected to Angle Two software was used to accurately target regions of the brain. After leveling the skull both in the ML and AP directions (within + /− 0.05 mm), the LHb was targeted using empirically determined coordinates and were verified using diI injections prior to any stereotaxic surgery experiments (from bregma, AP = −1.30, DV = −2.85, ML = ±0.4 mm). For all adeno-associated virus (AAV) experiments targeting the lateral habenula, 30 nL of virus was used per side. For chemogenetic behavior assays AAV-Ef1α-DIO-hM3D-mCherry (serotype 2/9) was used for gain-of-function experimental animals, AAV-Ef1α-flex-eGFP (serotype 2/9) was used for controls. All viruses were titered to at least 10^11^ viral particles/uL and generated in-house by the Neuroconnectivity Core at Baylor College of Medicine. Peri-operative and post-operative meloxicam analgesic at dosage of 5 mg/kg was administered in accordance with approved IACUC protocol (AN-5596). All *ex vivo* and *in vivo* experiments were initiated 2 weeks after surgery.

### *Post hoc* perfusion and tissue dissection

At a minimum of two-weeks following viral injection, mice were anesthetized using isoflurane and were transcardially perfused with PBS followed by 4% PFA (diluted using 16% paraformaldehyde EM Grade No. 15710 Electron Microscopy Sciences). For cFos staining, mice were injected with CNO 1 h prior to perfusion. Brains were extracted and drop-fixed in 4% PFA overnight at 4°C, followed by cryoprotection for 2 days in 30% sucrose in PBS at 4°C. Cryoprotected brains were then embedded and frozen in O.C.T. (Fisher HealthCare No. 4585) and stored at −80°C until sectioning. Brains were sliced coronally in the anterior to posterior direction on a cryostat (Leica CM1860) at 40 μm and every third section was collected for analysis. Slices were either separated for IHC antibody staining or directly mounted onto slides using DAPI Fluoromount-G (Southern Biotech, 0100-20).

### Immunohistochemistry

For immunohistochemistry, 40 μm floating slices were first rinsed for 5 min in PBS. They were then blocked for 1 h in 10% donkey or horse serum in PBS followed by primary antibody incubation (rabbit anti-cFos, abcam, 1:1000) in blocking solution at 4°C for 48 h. Secondary antibody incubation (647 goat α-rabbit, #A21244, Lot 2390713, LifeTechnologies, 1:500) was performed in blocking solution with 0.1% Triton X-100 at room temperature for 2 h. Sections were washed between incubations with PBS containing 0.1% Triton X-100. Sections were then mounted onto slides using DAPI Fluoromount-G (Southern Biotech, 0100-20). Images were acquired using a Leica SPX8 microscope at 10 or 20× magnification. For cFos detection, 3–4 images were taken of bilateral LHb regions (identified using the Allen Brain Atlas) across different Bregma coordinates, with at least three animals quantified for biological reproducibility. The fluorescent signals were quantified per LHb *post hoc* using Imaris.

### Ex-vivo electrophysiology

To test the effect of CNO on DREADD receptor expressing neurons, acute brain slices containing the lateral habenula were prepared from 12- to 16-week-old animals expressing mCherry-tagged hM3D. Animals were anesthetized with isoflurane and perfused with cold artificial cerebrospinal fluid (ACSF) solution pH 7.35, 305 mOsm, containing 128 mM NaCl, 3 mM KCl, 1.25 mM NaH_2_PO_4_, 2 mM CaCl_2_, 1 mM MgCl_2_, 10 mM Glucose, 25 mM NaHCO_3_. Brains were rapidly removed and transferred into sucrose-based cutting solution pH 7.35 containing: 85 mM NaCl, 2.5 mM KCl, 1.25 mM NaH_2_PO4-H_2_O, 0.5 mM CaCl_2_, 7 mM MgCl_2_, 13 mM Ascorbic Acid, 75 mM Sucrose, 25 mM Glucose, 25 mM NaHCO_3_ and continuously bubbled with 5% CO_2_/95% O_2_. 300 μm thick coronal brain slices were prepared using a Leica VT1200 vibratome and placed for recovery for at least 15 min at 34°C in 5% CO_2_/95% O_2_ bubbled ACSF solution. They were then gradually lowered to room temperature and allowed to acclimate for at least 20 min before recording. For recording, slices were transferred into a chamber continuously perfused with aCSF at 2 mL/min at room temperature. Neurons were identified by 900 nm IR-DIC (Olympus BX50WI) and fluorescent imaging using a Slice Scope Pro 6000 (Scientifica) platform, equipped with Thorlabs DC4100 470-nm or 565-nm LED illumination with 49002-ET-EGFP (FITC/Cy2) or HcRed1 emission filters (Chroma Technology), respectively. Recordings were obtained using an Axon MultiClamp 700B amplifier digitized at 10 kHz (Axon Digidata 1440A). Recording electrodes (3–5 MΩ) were fabricated from borosilicate glass microcapillaries (outer diameter, 1.5 mm) with a micropipette puller (Sutter Instruments). The internal (pipette) solution contained 120 mM K-gluconate, 10 mM KCl, 4 mM Mg-ATP, 10 mM Na_2_ phosphocreatine, 0.4 mM Mg-GTP, 10 mM HEPES, 1 mM EGTA, pH adjusted to 7.3 with KOH, 300 mOsm. Cells with a series resistance of less than 30 MΩ and <25% change for duration of the experiment were used for analysis. Cells were prerecorded in current clamp mode for at least 10 min, then CNO (10 uM) was applied to the bath perfusion. To estimate the total number of firing and non-firing cells with or without DREADD activation, a subset of cells (2–3 per brain slice) was recorded for at least 10 min each, before, and 1–2 more cells within the same slice—after CNO application. Total number of recordings: 38 cells, from 10 slices, 6 animals. In all recordings changes in resting voltage and firing mode were monitored online and analyzed using pClamp 10.7 software (Molecular Devices). Cells were defined as silent (non-firing) if no APs were detected over 10 min of the recording. Cells were classified as burst firing if they showed burst APs at any time during the recording.

### Novel object recognition with chemogenetic manipulations and ketamine rescue

The investigators were blinded to the experimental condition of the mice for the behavioral tests. Novel object recognition (NOR) was performed as previously described (Chen et al., 2019) with minor modifications. Prior to initiation of testing, mice were habituated to a black Plexiglas rectangular chamber (31 × 24 cm, height 27 cm) for 20 min under dim ambient light for 3 days. Once habituated, mice were advanced to novel object testing.

#### Serial NOR with chemogenetic excitation and washout

Mice were subject to 3 consecutive iterations of a 2-day testing protocol. On the first day of each 2-day session (i.e., days 1, 3, and 5 of the total timeline), mice were allowed to explore two identical objects in the testing chamber for 10 min, after which they immediately received i.p. injections of equal volumes of saline or CNO (3.5 mg/kg diluted in saline, Tocris 4963). CNO dosage determined based on proven efficacy in previous works (Moss et al., 2025). For the first NOR task, saline was administered to establish baseline memory function. For the second NOR task, CNO was administered to assess impact of chemogenetic excitation on memory function. For the third, saline was administered to assess memory after CNO washout. On the second day of each 2-day session (i.e., days 2, 4, and 6 of the total timeline), mice were again placed in the testing chamber and presented with one object used on the previous day (familiar object) and a novel object for 10 min. Objects presented to mice in NOR test 1 (days 1 and 2, saline injection) were not reused for consecutive tests. Objects presented in NOR test 2 (days 3 and 4, CNO injection), were reused in NOR test 3 (days 5 and 6, washout saline injection) to test whether these particular objects were inherently more difficult to discriminate.

#### Serial NOR with chemogenetic activation at distinct timepoints

Mice were subject to 3 consecutive iterations of a 2-day testing protocol. On the first day of each 2-day session (i.e., days 1, 3, and 5 of the total timeline), mice were allowed to explore two identical objects in the testing chamber for 10 min, after which they received i.p. injections of equal volumes of saline or CNO (3.5 mg/kg diluted in saline, Tocris 4963) at distinct time points after training. To study early memory consolidation, injections were performed directly after training. To assess late memory consolidation, injections were performed 6 h after training. To assess long term memory storage, injections were performed directly before testing (24 h). On the second day (24 h), test mice were again placed in the testing chamber and presented with one object used on the previous day (familiar object) and a novel object for 10 min. Objects for each NOR test were not reused for consecutive tests.

#### Serial NOR with chemogenetic activation and ketamine rescue

Mice were subject to 3 consecutive iterations of a 2-day testing protocol. On the first day of each 2-day session (i.e., days 1, 3, and 5 of the total timeline), mice were allowed to explore two identical objects in the testing chamber for 10 min, after which they received i.p. injections of equal volumes of saline, CNO (3.5 mg/kg diluted in saline, Tocris 4963) or CNO and ketamine (3.5 mg/kg CNO + 10 mg/kg ketamine) immediately after training. On the second day (24 h), test mice were again placed in the testing chamber and presented with one object used on the previous day (familiar object) and a novel object for 10 min. Objects for each NOR test were not reused for consecutive tests.

#### Analysis and object selection

For all NOR tests, objects were selected that had similar heights and volumes, but different shapes and appearances. Exploration of the objects was defined as sniffing (with nose contact or head directed to the object) within a 2 cm radius of the objects. Discrimination Index (DI) was computed as (Novel Object Exploration Time − Familiar Object Exploration Time/Total Exploration Time) × 100.

### Cocaine conditioned place preference with chemogenetic manipulations

The investigators were blinded to the experimental condition of the mice for the behavioral tests. Cocaine CPP, performed as previously described (Huang et al., 2016), was assessed over 6 days using an unbiased procedure and a standard two-chamber CPP apparatus (Ugo Basile, Varese, Italy). Animal behavior was videotaped with an overhead camera and analyzed by ANY-maze software (Stoelting, Wood Dale, IL). The difference in time spent in the cocaine-paired side versus the saline-paired side was calculated as the CPP score. On day 1, a mouse was placed in the chamber with the doors removed for a 30 min pre-training test and the baseline preference was calculated. On the following 4 days, training sessions were performed once a day. On alternate days, mice were given injections of cocaine (10 mg/kg, i.p.) or equivalent volume of saline (0.9% NaCl, i.p.) immediately before being confined to the cocaine-paired or saline-paired chamber for 30 min. After the pairing, mice were then injected with CNO (3.5 mg/kg diluted in saline) and then returned to their home cages. On day 6, a test session identical to the pre-training test was conducted to determine the CPP scores.

### Contextual and auditory fear conditioning with chemogenetic manipulations

Tests were performed on adult *Vglut2-Cre* littermates (<6 month age) by an experimenter blinded to the experimental conditions as previously described (Costa-Mattioli et al., 2007) with minor modifications. Mice were handled for 5 days before the start of the experiment. They were habituated to two distinct contexts (here named A and B) for 20 min for 3 days. After 3 days of habituation, mice were trained in chamber A. Training consisted of a 2 min period of acclimatizing to the context, followed by two pairings of a tone (2,800 Hz, 85 dB, 30 s) with a co-terminating foot-shock (0.7 mA, 2 s). The mice remained in the chamber for an additional 1 min after the end of the last pairing, after which they received an i.p. injection of CNO (3.5 mg/kg) and were returned to their home cages. Contextual fear conditioning was assayed 1 h and 24 h after training by replacing the animals in the conditioning context (chamber A) for a 5 min period, during which the incidence of freezing (immobile except for respiration) was recorded. The tests of auditory fear conditioning consisted of a 2 min acclimatizing period to the context (chamber B; pre-CS period), followed by a 3 min presentation of the same tone (2,800 Hz, 85 dB, 30 s) (CS). Mice were returned to their cages 30 s after the end of the tone. For all tests, each mouse was judged at 5 s intervals as either freezing or not freezing. Real-time video was recorded and was analyzed using FreezeView as we previously described (Chen et al., 2019). Data were expressed as the percent of 5 s intervals in which freezing was observed. Tests of responses to the training context (chamber A) and to the tone (chamber B) were performed in a counterbalanced manner.

### Anxiety and depression assays

#### Open field test

Tests were performed on adult *Vglut2-Cre* littermates (<6 month age) by an experimenter blinded to experimental conditions as previously described. Mice were allowed to freely explore a Noldus Phenotyper arena and monitored for 10 min using Noldus Ethovision Software (XT 16; https://www.noldus.com/ethovision-xt). Video tracking was used to evaluate distance traveled and time spent in the center of the arena.

#### Forced swim test

Tests were performed on adult *Vglut2-Cre* littermates (<6 month age) by an experimenter blinded to experimental conditions as previously described (Fan et al., 2023). Briefly, mice were placed in a transparent cylindrical container filled with water maintained at 23–25°C for a total test time of 6 min. Mice were recorded with ANY-Maze and immobility, defined as animals remaining floating or motionless with only small and necessary movements for keeping balance, was manually scored.

### Quantification and statistical analysis

GraphPad Prism 10.2.3 (GraphPad Software, Inc., La Jolla, CA, USA) was employed for all statistical analyses and figure generation. All data were evaluated for normality and homogeneity of variances before performing statistical analyses. Differences in object investigation times were evaluated using One-way ANOVA with Sidak multiple comparisons for parametric data or Kruskal–Wallis tests with multiple comparisons for non-parametric data without assuming equal variance. Discrimination indices were evaluated using unpaired Student’s *t*-tests for parametric data and Mann–Whitney tests for non-parametric data. CPP score differences were evaluated with paired Student’s *t*-tests. Contextual and auditory conditioning freezing percentages were evaluated using Mann–Whitney tests. Open field assays monitoring locomotion and duration in the center, as well as immobility time on forced swim tests were evaluated using Mann–Whitney tests. Cell firing phenotypes before and after CNO bath application in electrophysiological recordings were evaluated using Chi-square tests. Throughout all experiments, a *p*-value of less than 0.05 was considered indicative of significant changes. Sample sizes were determined based on established literature standards, with *post-hoc* analyses of all significant results powered >0.75. Additional detailed statistical information, including the exact value of n and its representation for each experiment, is included in the text.

## Results

### Chemogenetic activation of LHb glutamatergic neurons disrupts memory

To investigate the cell type-specific effects of LHb glutamatergic neuronal activity in memory function, we used a conditional viral approach in *vGlut2-Cre* mice to express an mCherry-tagged Designer Receptor Exclusively Activated by Designer Drugs (DREADD) hM3D, or a GFP control virus in the LHb (Figure 1a). Following systemic administration of clozapine-N-oxide (CNO), which allows for controllable activation of the hM3D-expressing glutamatergic LHb neurons, we observed a significant increase in cFos expression in the hM3D cohort compared to GFP-injected controls (Figures 1b,c). Thus, CNO induces recruitment of hM3D-expressing glutamatergic LHb neurons. To further validate hM3D viral expression and function, we performed visually guided electrophysiological recordings in *ex vivo* brain slices from individual mCherry-expressing LHb cells with and without CNO. Although not all recordings showed consistent increases in firing after CNO, average firing rates trended towards increased action potential firing in mCherry labeled cells, consistent with a network excitatory response due to hM3D-mediated responses to CNO (Figures 1d,e).

Previous work on excitatory forebrain inputs to glutamatergic LHb neurons has revealed that activation of LHb-projecting basal forebrain cells drives a reflexive aversion that is associated with impaired memory (Swanson et al., 2022). To further investigate whether the glutamatergic neurons within the LHb may play a larger role in memory function, we chemogenetically activated the glutamatergic LHb neurons and tested mice in a serial novel object recognition assay (serial NOR) (Figure 1f). After stereotaxically delivering conditional viruses expressing mCherry-tagged hM3D or a control GFP reporter in *Vglut2-Cre* mice, we began behavioral experimentation. Each mouse completed three consecutive NOR tests, with each test following a two-day protocol, resulting in 6 days of total behavioral testing per mouse. Each individual NOR test consisted of a two-day protocol where mice were exposed to two identical objects on training day, after which they were immediately injected with CNO to activate the hM3D-expressing vGlut2+ LHb neurons, or injected with saline as a control. The next day, one of the objects was replaced with a novel object, and we recorded the amount of time spent investigating the familiar vs. novel objects (Figure 1f). We then measured their ability to discriminate between the objects using a discrimination index. For the first set of objects, all mice were injected with saline post-training to validate proper memory function at baseline for all animals. Both the hM3D-expressing and GFP-expressing cohorts of mice spent a significantly greater time investigating the novel object compared to the familiar one, with no significant differences observed in discrimination ability between cohorts (Figure 1g), suggesting a baseline ability to remember the previously exposed familiar object. Once we confirmed that all mice were appropriately able to recognize familiar objects, we exposed them to a new set of objects and injected all mice (including GFP controls) with CNO after training. We then recorded their investigation times of the new set of familiar and novel objects to assess the effect of chemogenetic activation of LHb glutamatergic neurons on memory function. Interestingly, the activation of LHb glutamatergic neurons after training resulted in memory deficits as shown by a lack of preference between novel and familiar objects on test day, and a significantly lower discrimination index in the hM3D cohort compared to the GFP controls (Figure 1h). Finally, to rule out the possibility that the second pair of objects was inherently more difficult to learn than the first, we repeated the test with a second set of objects after CNO washout. All mice were able to recognize the familiar object, showing a significant preference for the novel object, and once again, we observed no significant discrimination ability differences between cohorts, indicating that the memory deficit observed with CNO administration was specifically driven by activation of LHb glutamatergic neurons (Figure 1i). Taken together, these data show that activation of LHb glutamatergic neurons post-training in novel object recognition tasks is sufficient to impair recognition of previously exposed familiar objects.

### Excitation of LHb glutamatergic neurons disrupts memory consolidation

To investigate how the activity of glutamatergic LHb neurons influences memory consolidation and long-term memory storage, we activated these cells at different stages of the memory storage process. Importantly, given the transient nature of chemogenetic manipulations, we were able to perform repeated LHb activation experiments in the same mice, allowing us to use these cohorts as their own internal controls to account for any individual variability in performing associative memory tasks. By activating the glutamatergic LHb neurons at distinct timepoints between training and testing in the novel object task, we sought to further dissect the temporal necessity of LHb activity in maintaining proper memory function. To this end, we delivered either CNO or saline to hM3D-expressing and GFP-expressing control mice at distinct time points between training sessions that corresponded with specific memory stages: injection immediately after training, 6 h and 24 h post-training. Injection within the first hour of training tests early phase consolidation, injection 6 h post-training tests late phase consolidation, and injection 24 h post-training tests long-term memory storage (Figure 2a). Notably, these timepoints were chosen intentionally based on prior memory-association studies (Nader et al., 2000; Costa-Mattioli et al., 2007; Igaz et al., 2002; Grecksch and Matthies, 1980). As above, we measured time spent with novel and familiar objects on test day to assess memory function, followed by calculating a discrimination index for each mouse to assess its ability to discriminate between novel and familiar objects.

When CNO was systemically administered immediately after training, corresponding to the early phase of consolidation, the chemogenetically activated cohort expressing hM3D displayed no preference for the familiar or novel objects based on investigation time, with a significantly reduced discrimination ability from their own saline-injected baselines as well as the GFP expressing control cohort (Figure 2b). In contrast, CNO-injection 6 h post-training, which is outside of the early consolidation window, had no major effect since all mice showed a significant preference for the novel object with saline and CNO injections. However, the hM3D cohort showed a reduced ability to discriminate compared to their own saline-injected baselines and GFP controls (Figure 2c). Hence, LHb activation affects mostly long-term object recognition memory consolidation. Finally, when we tested the effect of chemogenetically activated LHb glutamatergic cells 24 h post-training, corresponding to the long-term memory storage stage, while the hM3D-activated mice did show a lower discrimination ability than their GFP control counterparts, they were still able to maintain a significant preference for the novel object compared to the familiar one (Figure 2d). These data indicate that the activation of glutamatergic LHb neurons can drive memory deficits in a limited temporal window post-training, corresponding to the memory consolidation stage, and that this influence becomes weaker with time from exposure. Importantly, these data represent the first characterization of a critical temporal window after associative training in which hyperactivation of glutamatergic LHb neurons affects memory.

### Activation of LHb glutamatergic neurons disrupts reward association, but does not impact fear memory

While we had established a major role for the activity of glutamatergic LHb neurons in the preservation of proper memory function in a neutral recognition task, we were intrigued by the potential for LHb activity to influence valence-associative memory as well. Especially given the nature of the LHb as a prominent region that regulates mood, emotion, reward, and aversion. Thus, we next sought to test how glutamatergic neural activation in the LHb could impact reward and long-term fear memories.

An established potent stimulus used for reward conditioning in mice is cocaine. To selectively test reward-associated memory, we used a cocaine-conditioned place preference assay (CPP) in which mice were exposed to two halves of a chamber and trained on four alternating days to associate one half with a positive stimulus (cocaine) and the other half with a neutral stimulus (saline). Prior to exposure to the arena, a baseline preference is taken to account for any inherent preference each mouse has for either side. During the training phase, cocaine or saline is injected immediately prior to a 30-min exposure period in the, respectively, associated half of the arena, restricting the mouse’s movement to only that half of the arena by closing a door between the two sides of the chamber. After the 4 days of training are complete, the mouse is then allowed to freely explore both sides of the chamber and preference is recorded as a CPP score, which represents the difference between time spent in the cocaine-associated half vs. the saline-associated half. To test whether chemogenetic activation of LHb glutamatergic neurons could impair reward memory, we systemically delivered CNO into hM3D-expressing and GFP-expressing control mice within the consolidation time window post-training on all 4 days of associative training (Figure 3a). As expected for mice with intact reward-associated memory, we saw a significant increase in CPP score in the GFP controls, indicating that these mice showed an increased preference for the cocaine-associated half of the arena. However, the hM3D-expressing mice displayed a striking lack of preference for the cocaine-associated half of the chamber, indicating a failure to store a reward-associated memory with glutamatergic neuronal activation in the LHb (Figure 3b). Thus, the activity of glutamatergic LHb neurons is sufficient to impair reward-associated memory.

Given the significant impact of glutamatergic LHb neuronal activation in impairing reward associated memory, we next sought to understand how negative valence-associative memory could be impacted by LHb activation. Especially due to its prominence as an aversion center, we hypothesized that LHb activation could produce a significant impact on aversively tagged memories. To assess negative valence-associative memory, we used a classical fear conditioning paradigm (Figure 3c). After 3 days of habituation to the shock chambers, we placed the hM3D and GFP-expressing mice into the chamber and measured baseline freezing for 2 min, after which we presented two pairings of an auditory tone that terminated with an electric foot shock (0.7 mA, 2 s). Immediately following training, we administered CNO to both groups to artificially activate the glutamatergic LHb neurons in hM3D-expressing mice. To assay contextual fear conditioning, we returned the mice to the same shock chambers the next day and recorded freezing time for 5 min to assess contextual fear conditioning. To assay cued fear conditioning, we placed the mice in a novel chamber allowing for a 2-min habituation period, followed by a 3-min presentation of the conditioned auditory tone, and measured freezing response. Freezing responses were recorded as percentages of the time measured. In both the hM3D and GFP-expressing cohorts, we observed a significant freezing response to the context and cues associated with shock training, with no significant differences between hM3D-expressing mice and the GFP-expressing controls (Figure 3d). These data indicate that chemogenetic activation of glutamatergic LHb neurons after fear conditioning is not sufficient to disrupt fear memory. In sum, we find that altered LHb glutamatergic neuronal activity during consolidation affects the preservation of positive valence memories, but not aversively tagged ones.

### Ketamine rescues memory deficits in mice with chemogenetically activated glutamatergic LHb neurons

Given that LHb hyperactivity has been observed as a potential biomarker of depression and has been associated with anxiety and stressful states, we next asked whether the memory deficits driven by LHb glutamatergic neuronal activation were part of a larger depression or anxiety phenotype driven by LHb hyperactivity. In an open field assay for anxiety, we found that neither the hM3D-expressing or GFP-expressing mice showed differences in locomotion or time spent in the center of the arena (Supplementary Figures 1a,b). Using the forced swim test to assess depression behavior, neither cohort showed differences in immobility time (Supplementary Figure 1c) or major changes in depression-associated burst firing electrophysiological phenotypes in the vGlut2+ LHb cells (Supplementary Figure 1d). These data show that activation of glutamatergic LHb neurons during consolidation can impair memory independent of an anxious or depressive state, suggesting that the LHb may have an evolutionarily conserved role in memory preservation that is vulnerable to activity modulation.

**Figure 4:**
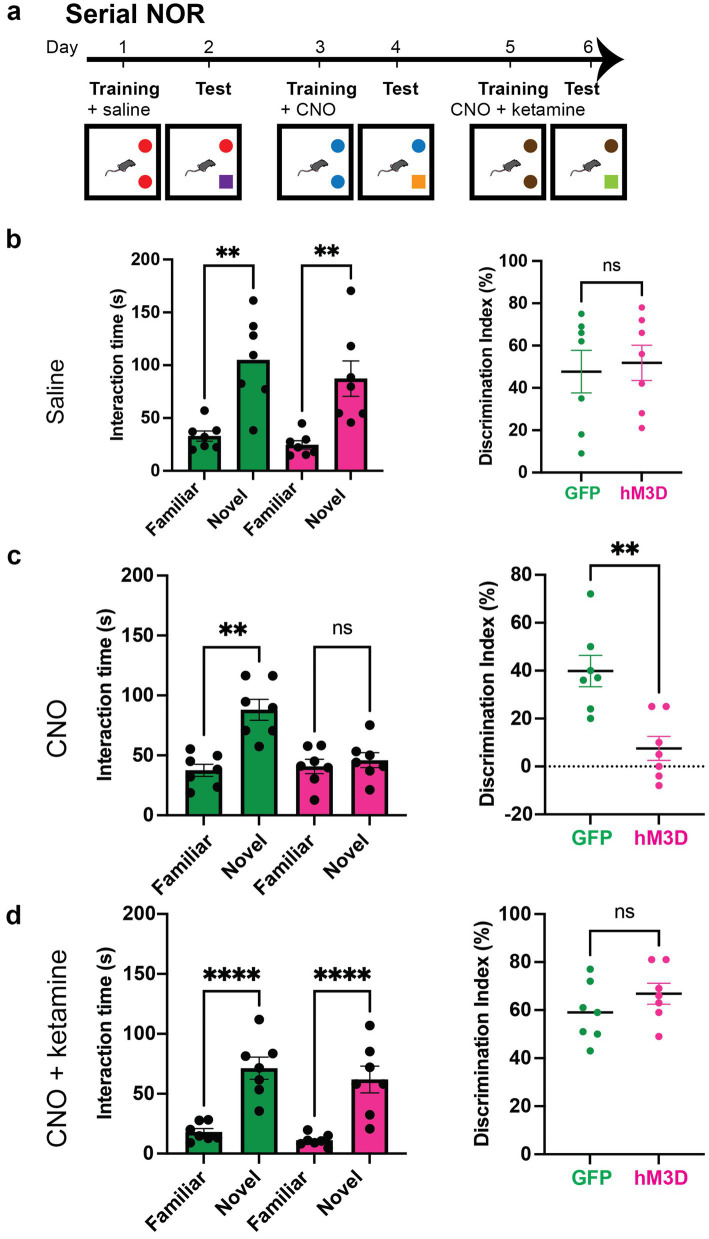
Ketamine rescues memory impairments caused by chemogenetic activation of vGlut2+ LHb neurons. **(a)** Experimental paradigm for serial novel object recognition tasks with chemogenetic activation of vGlut2+ LHb neurons. GFP and hM3D mice are injected with saline, CNO or mixture of CNO and ketamine post-training and tested the following day for ability to discriminate between familiar and novel objects. **(b)** Time spent with familiar and novel objects (left) measured in GFP control (*n* = 7) and hM3D experimental (*n* = 7) groups injected with saline post-training. Statistical significance between novel and familiar object investigation time calculated by Kruskal–Wallis test with multiple comparisons (GFP familiar vs. novel *p* = 0.0062, hM3D familiar vs. novel *p* = 0.0013, alpha = 0.05). Discrimination index (right) on test day of novel object recognition. Statistical significance between DI hM3D and DI GFP calculated by unpaired Student’s *t*-test (*p* = 0.7572, alpha = 0.05). **(c)** Time spent with familiar and novel objects (left) measured in GFP control (*n* = 7) and hM3D experimental (*n* = 7) groups injected with CNO post-training. Statistical significance between novel and familiar object investigation time calculated by Kruskal–Wallis test with multiple comparisons (GFP familiar vs. novel *p* = 0.0034, hM3D familiar vs. novel *p* = 0.8091, alpha = 0.05). Discrimination index (right) on test day of novel object recognition. Statistical significance between DI hM3D and DI GFP calculated by Mann–Whitney test (*p* = 0.0064, alpha = 0.05). **(d)** Time spent with familiar and novel objects (left) measured in GFP control (*n* = 7) and hM3D experimental (*n* = 7) groups injected with mixture of CNO and ketamine post-training. Statistical significance between novel and familiar object investigation time calculated by Kruskal–Wallis test with multiple comparisons (GFP familiar vs. novel *p* = 0.0055, hM3D familiar vs. novel *p* = 0.0006, alpha = 0.05). Discrimination index (right) on test day of novel object recognition. Statistical significance between DI hM3D and DI GFP calculated by Mann–Whitney (*p* = 0.2692, alpha = 0.05).

Although chemogenetically activating glutamatergic LHb neurons does not drive depression-like behavior, studies have shown that ketamine acts preferentially on hyperactivated LHb neurons to mediate antidepressant effects (Yang et al., 2018a; Ma et al., 2023). Ketamine is a known NMDA receptor antagonist, and notably, previous work has shown that hM3D-mediated chemogenetic activation in the hippocampus confers heightened sensitivity to ketamine action (Chen et al., 2024). Given these findings, we asked whether the memory deficits observed after chemogenetically activating LHb glutamatergic neurons could be rescued by ketamine administration. To answer this question, we injected a new cohort of *vGlut2-Cre* mice for conditional viral expression of mCherry-tagged hM3D or GFP control virus in the LHb. After 2 weeks post-surgery to allow for recovery and viral expression, we tested the cohort on a new set of serial NOR tasks. Each novel object task consisted of a two-day paradigm where mice were exposed to two identical objects on training day, after which they received an injection of saline (control), CNO (hM3D activation), or CNO and ketamine mixture (rescue). Importantly, single doses of ketamine are known to produce long lasting effects. Thus, we only administered the CNO and ketamine mixture for the final NOR task, after which mice were removed from behavioral testing. The day after training, one of the previously exposed objects was replaced with a novel object and we recorded the amount of time spent investigating the familiar and novel objects (Figure 4a). As expected, all animals were able to discriminate and recognize objects appropriately when injected with saline, and this ability was abolished with CNO injection post-training (Figures 4b,c). After CNO washout, the same mice were trained on a novel set of objects and injected with a mixture of CNO and ketamine after training. Strikingly, all mice were able to recognize the familiar object, showing a significant preference for the novel object without significant differences in discrimination ability between the experimental and control mice (Figure 4d). These results indicate that ketamine administration was sufficient to rescue the chemogenetically-induced memory deficits seen in the hM3D-expressing mice. Taken together, our findings indicate that activation of glutamatergic LHb neurons during memory consolidation is sufficient to disrupt memory in the absence of an anxiety or depressive state. And that this disruption of memory can be rescued by ketamine, suggesting glutamatergic LHb neurons play a critical role in memory consolidation through a NMDA receptor-mediated mechanism.

## Discussion

Here we used a chemogenetic approach to selectively activate glutamatergic neurons within the LHb to evaluate their role in guiding higher order cognitive and memory processing. Using a serial NOR task paradigm, we identified a critical role for glutamatergic LHb neuronal activity in the preservation of neutral memories. We performed the first temporal characterization study of this effect and identified a critical time window during which memory is vulnerable to LHb glutamatergic neuronal activation. Using cocaine CPP and fear conditioning, we found that both neutral and reward associative memories are disrupted by LHb glutamatergic neuronal activation during consolidation, while fear memory remained intact. Finally, we found that the memory impairments produced by LHb glutamatergic neuronal activation during consolidation were independent of an anxious or depressive state, and that these memory deficits could be rescued by ketamine administration.

The lateral habenula has been widely studied in the context of reward/aversion processing and increasingly in the context of major depressive disorder (MDD) (Hu et al., 2020). Notably, fMRI studies identified the LHb as a significant region of increased connectivity in MDD patients compared to healthy controls (Gosnell et al., 2019a). In rodent models of depression, neuronal burst firing has been identified as a potential biomarker of depressive state that can be ameliorated by the NMDA antagonism of ketamine (Yang et al., 2018a). Given these findings, we hypothesized that hyperactivation of the glutamatergic LHb would be sufficient to drive depression, and that our memory disruption findings could explain the cognitive impairments that are often seen in tandem with depression. Surprisingly, we found that while there are clear memory impairments with chemogenetic activation of the LHb, this manipulation does not drive depression. This may be due to the type and level of stimulation applied to the LHb. For example, most models of depression such as chronic restraint stress (CRS) or social defeat models involve chronic stressful treatments of the animal, and perhaps this chronic nature is necessary to drive plasticity in the endogenous activity of LHb neurons (Chiba et al., 2012; Harro, 2019). Although acutely induced models of depression exist, they rely upon optogenetic actuators whose mechanism of activation is inherently different from chemogenetic ones. One such optogenetic model uses an inhibitory halorhodopsin to induce depression that depends on a post-inhibitory rebound effect to cause burst firing (Yang et al., 2018a). However, our electrophysiological recordings reveal no such increase in percentage of burst firing neurons. We posit that while hM3D-mediated activation can impair memory, the potency of this activation does not reach the sufficient level achieved by optogenetic actuators or chronic stress treatments to drive depression.

The striking effect of LHb glutamatergic activation on memory consolidation begs the question of whether LHb activity could be contributing to the cognitive deficits that often accompany depression in patients (Culpepper et al., 2017). Depressed patients have been shown to experience significant cognitive deficits in executive function, memory and attention that persist even during remission, indicating that there are plasticity mechanisms at play in depressive states that alter cognitive processing (Rock et al., 2014). Given the known link between habenular network hyperactivity and depression (Li et al., 2013; Gosnell et al., 2019b), our findings support the idea that disrupted LHb activity may play a direct role in mediating these cognitive impairments through maladaptive neural plasticity. Notably, the intersection between depression and memory impairment remains an understudied topic in behavioral neuroscience. Our findings demonstrate that memory consolidation is sensitive to LHb modulation, underscoring the need for further investigations into the circuit, molecular and signaling mechanisms by which LHb activity modulates cognitive function. Such studies will be crucial for understanding the underlying mechanisms by which LHb activity dysfunction contributes to cognitive deficits observed in depressed patients.

Moreover, studies have implicated increased LHb network connectivity as a potential indicator for treatment response in depressed psychiatric patients (Gosnell et al., 2019a). Our finding that ketamine rescues memory deficits induced by LHb hyperactivation points to a potential role for cognitive function as predictive indicator of therapeutic efficacy. These results suggest that incorporating cognitive assessments into treatment planning in the clinic may enhance patient outcomes, allowing providers to better identify the patients most likely to benefit from treatment with a rapid-acting antidepressant like ketamine.

One of the most interesting and unexplained aspects of our study is the exact mechanism underlying how LHb activity impacts on memory. Anatomical and functional tracing approaches have identified several prominent downstream regions such as the dopaminergic ventral tegmental area (VTA) and serotonergic raphe nuclei that could be mediating the influence of LHb activity on memory (Xin et al., 2022; Baker et al., 2022). Notably, dopaminergic and serotonergic signaling systems have been shown to modulate different types of spatial and reward-associated memory, and the release of these neurotransmitters are both directly inhibited by LHb connectivity (Seyedabadi et al., 2014; Glykos and Fujisawa, 2024; Hu et al., 2020; Srivastava et al., 2023). Even more intriguingly, the LHb has no known projections to the hippocampus, which would be the most obvious canonical region to explore in a memory context, suggesting that LHb activity must be indirectly influencing memory through intermediary connections. Though previous work has implicated a role for synchronized LHb and hippocampal oscillations in the formation of memory (Goutagny et al., 2013; Aizawa et al., 2013), it remains an open question as to how this synchrony is generated and impacted by activity manipulations. Potential future investigations into the molecular substrates of memory consolidation, such as transcriptional regulation of protein synthesis (Buffington et al., 2014; Costa-Mattioli et al., 2007; Grecksch and Matthies, 1980), may provide further mechanistic insights. Finally, additional studies have implicated a critical role for medial prefrontal cortex (mPFC) input to the LHb in memory formation and retrieval (Mathis et al., 2017), and perhaps our work here can provide further insight on how this effect is mediated through LHb activity changes.

The critical temporal window of memory vulnerability to LHb activity suggests that while the LHb may not be a primary memory hub, abnormal activity can cause a major disruption of memory due to its highly interconnected nature and its low level of baseline activity (Hu et al., 2020). This suggests that the LHb may be an early gate of information processing which shunts information to more basic behavioral outputs without engaging higher order cognition and learning. This may point to a critical role of LHb activity in adapting behavior to prioritize escape or avoidance over higher order processes like cognition, or learning and memory. In fact, previous work has revealed that optogenetic stimulation of LHb activity directly after novel object training can cause a reflexive aversive state that bypasses memory (Swanson et al., 2022). These data combined with our findings that early hyperactivation in the post-training period of our behavioral assays was sufficient to impair memory expression are highly suggestive of the LHb acting as an early modulator of information processing.

The task-specific nature of the memory impairments caused by hyperactivation of LHb glutamatergic neurons reveals another level of complexity to the role of LHb activity in memory. Although both cocaine and electric foot shocks are potent reward and punishment stimuli respectively, the LHb hyperactivation performed in our study only yielded a significant impairment in reward and neutral associative memory, while leaving fear memory intact. One potential reason for this is that aversively tagged memories may rely on LHb activation as part of the memory storage process, and by increasing the activity of the LHb after training, we did not introduce a sufficient change in the LHb activity network to alter memory expression. It would be important to study the effects of inhibiting these glutamatergic LHb cells to fully dissect whether this is the case. For example, if memory is impacted by LHb glutamatergic connections to downstream neuromodulatory centers, then activating versus inhibiting these pathways may have opposing effects on downstream information processing, leading to divergent behavioral outcomes. Supporting this, a previous study found that inhibiting glutamatergic input to the LHb is sufficient to impair memory encoding and retrieval, but not consolidation, in spatial memory tasks (Mathis et al., 2015). This suggests that the behavioral impact of LHb activity depends on the direction of manipulation, potentially through the engagement of distinct neuronal connections. Another potential reason why LHb activation leaves fear memory intact is that fear memory is also processed through parallel circuits such as those involving the hippocampus and amygdala (Izquierdo et al., 2016). These circuits may be more hardwired and less susceptible to LHb-driven plasticity than those that encode for neutral and reward memory, making them less likely to be affected by LHb manipulations.

Perhaps the most interesting remaining question is the mechanism by which ketamine is rescuing memory impairments caused by activation of glutamatergic LHb neurons. Recent work focused on ketamine’s antidepressant actions in the LHb has pointed to an activation-dependent conformational change of the NMDA receptor that makes those receptors particularly susceptible to the antagonistic effects of ketamine (Ma et al., 2023; Chen et al., 2024). Perhaps by chemogenetically activating the glutamatergic LHb neurons, we induced the same types of conformational changes that allowed ketamine to neutralize the memory impairments. Other potential mechanisms may be through extrasynaptic NMDA antagonism acting on local microcircuits within the LHb or glial components that reduce the activity of the region (Zanos and Gould, 2018). Given the long-lasting effects that a single dose of ketamine can have in treating depression (Berman et al., 2000; Zarate et al., 2006), it would be compelling to study whether ketamine can also prevent memory deficits when administered prior to associative training, and to determine how long this protective effect can persist post-drug delivery. Although these questions await further investigations, ongoing molecular and temporal dissections of limbic circuit activity, such as those conducted in the present study, continue to build upon our evolving understanding of how distinct neural populations act in concert to coordinate complex cognitive behaviors like learning and memory.

## Data Availability

The raw data supporting the conclusions of this article will be made available by the authors, without undue reservation.

## Supplementary material

SUPPLEMENTARY FIGURE 1 hM3D activation of vGlut2+ LHb neurons does not drive anxiety or depression-associated phenotypes. **(a)** Differences in distance traveled during 10-minute open field exploration after CNO administration in hM3D and GFP control mice. Statistical significance calculated using Mann-Whitney test (*p* = 0.5556, alpha = 0.05). **(b)** Differences in time spent in center of arena during open field anxiety test between hM3D experimental (*n* = 5) and GFP control (*n* = 4) groups. Statistical significance calculated using Mann-Whitney test (*p* = 7302, alpha = 0.05). **(c)** Differences in time spent immobile during forced swim test depression assay between hM3D experimental (*n* = 5) and GFP control (*n* = 4) groups. Statistical significance calculated using Mann-Whitney (*p* = 0.9048, alpha = 0.05). **(d)** Number of vGlut2+ LHb cells exhibiting silent, single AP, and burst firing properties in ex vivo electrophysiological recordings of hM3D mice (*n* = 6) before and after bath application of CNO. Differences between control and CNO conditions evaluated using Chi-square test (Chi square = 0.918, df = 2, *p* = 0.632, alpha = 0.05).

## References

[ref1] Aizawa H. Yanagihara S. Kobayashi M. Niisato K. Takekawa T. Harukuni R.. (2013). The synchronous activity of lateral habenular neurons is essential for regulating hippocampal theta oscillation. J. Neurosci. 33, 8909–8921. doi: 10.1523/JNEUROSCI.4369-12.2013, PMID: 23678132 PMC6618841

[ref2] Andalman A. S. Burns V. M. Lovett-Barron M. Broxton M. Poole B. Yang S. J.. (2019). Neuronal dynamics regulating brain and behavioral state transitions. Cell 177, 970–985.e20. doi: 10.1016/j.cell.2019.02.037, PMID: 31031000 PMC6726130

[ref3] Baker P. M. Mathis V. Lecourtier L. Simmons S. C. Nugent F. S. Hill S.. (2022). Lateral Habenula beyond avoidance: roles in stress, memory, and decision-making with implications for psychiatric disorders. Front. Syst. Neurosci. 16:826475. doi: 10.3389/fnsys.2022.826475, PMID: 35308564 PMC8930415

[ref4] Berman R. M. Cappiello A. Anand A. Oren D. A. Heninger G. R. Charney D. S.. (2000). Antidepressant effects of ketamine in depressed patients. Biol. Psychiatry 47, 351–354. doi: 10.1016/s0006-3223(99)00230-9, PMID: 10686270

[ref5] Buffington S. A. Huang W. Costa-Mattioli M. (2014). Translational control in synaptic plasticity and cognitive dysfunction. Annu. Rev. Neurosci. 37, 17–38. doi: 10.1146/annurev-neuro-071013-014100, PMID: 25032491 PMC4721605

[ref6] Chen M. Ma S. Liu H. Dong Y. Tang J. Ni Z.. (2024). Brain region-specific action of ketamine as a rapid antidepressant. Science 385:eado7010. doi: 10.1126/science.ado7010, PMID: 39116252 PMC11665575

[ref7] Chen C. -J. Sgritta M. Mays J. Zhou H. Lucero R. Park J.. (2019). Therapeutic inhibition of mTORC2 rescues the behavioral and neurophysiological abnormalities associated with Pten-deficiency. Nat. Med. 25, 1684–1690. doi: 10.1038/s41591-019-0608-y, PMID: 31636454 PMC7082835

[ref8] Chiba S. Numakawa T. Ninomiya M. Richards M. C. Wakabayashi C. Kunugi H. (2012). Chronic restraint stress causes anxiety- and depression-like behaviors, downregulates glucocorticoid receptor expression, and attenuates glutamate release induced by brain-derived neurotrophic factor in the prefrontal cortex. Prog. Neuro-Psychopharmacol. Biol. Psychiatry 39, 112–119. doi: 10.1016/j.pnpbp.2012.05.018, PMID: 22664354

[ref9] Congiu M. Mondoloni S. Zouridis I. S. Schmors L. Lecca S. Lalive A. L.. (2023). Plasticity of neuronal dynamics in the lateral habenula for cue-punishment associative learning. Mol. Psychiatry 28, 5118–5127. doi: 10.1038/s41380-023-02155-3, PMID: 37414924 PMC11041652

[ref10] Costa-Mattioli M. Gobert D. Stern E. Gamache K. Colina R. Cuello C.. (2007). eIF2alpha phosphorylation bidirectionally regulates the switch from short- to long-term synaptic plasticity and memory. Cell 129, 195–206. doi: 10.1016/j.cell.2007.01.050, PMID: 17418795 PMC4149214

[ref11] Culpepper L. Lam R. W. McIntyre R. S. (2017). Cognitive impairment in patients with depression: awareness, assessment, and management. J. Clin. Psychiatry 78, 1383–1394. doi: 10.4088/JCP.tk16043ah5c, PMID: 29345866

[ref12] Fan Z. Chang J. Liang Y. Zhu H. Zhang C. Zheng D.. (2023). Neural mechanism underlying depressive-like state associated with social status loss. Cell 186, 560–576.e17. doi: 10.1016/j.cell.2022.12.033, PMID: 36693374

[ref13] Flerlage W. J. Simmons S. C. Thomas E. H. Gouty S. Cox B. M. Nugent F. S. (2024). Dysregulation of kappa opioid receptor neuromodulation of lateral habenula synaptic function following a repetitive mild traumatic brain injury. Pharmacol. Biochem. Behav. 243:173838. doi: 10.1016/j.pbb.2024.173838, PMID: 39067532 PMC11344655

[ref14] Glykos V. Fujisawa S. (2024). Memory-specific encoding activities of the ventral tegmental area dopamine and GABA neurons. eLife 12:RP89743. doi: 10.7554/eLife.89743, PMID: 38512339 PMC10957172

[ref15] González-Pardo H. Conejo N. M. Lana G. Arias J. L. (2012). Different brain networks underlying the acquisition and expression of contextual fear conditioning: a metabolic mapping study. Neuroscience 202, 234–242. doi: 10.1016/j.neuroscience.2011.11.064, PMID: 22173014

[ref16] Gosnell S. N. Curtis K. N. Velasquez K. Fowler J. C. Madan A. Goodman W.. (2019a). Habenular connectivity may predict treatment response in depressed psychiatric inpatients. J. Affect. Disord. 242, 211–219. doi: 10.1016/j.jad.2018.08.026, PMID: 30195174

[ref17] Gosnell S. N. Fowler J. C. Salas R. (2019b). Classifying suicidal behavior with resting-state functional connectivity and structural neuroimaging. Acta Psychiatr. Scand. 140, 20–29. doi: 10.1111/acps.13029, PMID: 30929253

[ref18] Goutagny R. Loureiro M. Jackson J. Chaumont J. Williams S. Isope P.. (2013). Interactions between the lateral habenula and the hippocampus: implication for spatial memory processes. Neuropsychopharmacology 38, 2418–2426. doi: 10.1038/npp.2013.142, PMID: 23736315 PMC3799061

[ref19] Grecksch G. Matthies H. (1980). Two sensitive periods for the amnesic effect of anisomycin. Pharmacol. Biochem. Behav. 12, 663–665. doi: 10.1016/0091-3057(80)90145-8, PMID: 7393961

[ref20] Groos D. Helmchen F. (2024). The lateral habenula: a hub for value-guided behavior. Cell Rep. 43:113968. doi: 10.1016/j.celrep.2024.113968, PMID: 38522071

[ref21] Harro J. (2019). Animal models of depression: pros and cons. Cell Tissue Res. 377, 5–20. doi: 10.1007/s00441-018-2973-0, PMID: 30560458

[ref22] Hu H. (2016). Reward and aversion. Annu. Rev. Neurosci. 39, 297–324. doi: 10.1146/annurev-neuro-070815-014106 27145915

[ref23] Hu H. Cui Y. Yang Y. (2020). Circuits and functions of the lateral habenula in health and in disease. Nat. Rev. Neurosci. 21, 277–295. doi: 10.1038/s41583-020-0292-4 32269316

[ref24] Huang W. Placzek A. N. Viana Di Prisco G. Khatiwada S. Sidrauski C. Krnjević K.. (2016). Translational control by eIF2α phosphorylation regulates vulnerability to the synaptic and behavioral effects of cocaine. eLife 5:e12052. doi: 10.7554/eLife.12052 26928234 PMC4786430

[ref25] Igaz L. M. Vianna M. R. M. Medina J. H. Izquierdo I. (2002). Two time periods of hippocampal mRNA synthesis are required for memory consolidation of fear-motivated learning. J. Neurosci. 22, 6781–6789. doi: 10.1523/JNEUROSCI.22-15-06781.2002, PMID: 12151558 PMC6758123

[ref26] Ilango A. Shumake J. Wetzel W. Scheich H. Ohl F. W. (2013). Electrical stimulation of lateral habenula during learning: frequency-dependent effects on acquisition but not retrieval of a two-way active avoidance response. PLoS One 8:e65684. doi: 10.1371/journal.pone.0065684, PMID: 23840355 PMC3695985

[ref27] Izquierdo I. Furini C. R. G. Myskiw J. C. (2016). Fear memory. Physiol. Rev. 96, 695–750. doi: 10.1152/physrev.00018.2015, PMID: 26983799

[ref28] Karamihalev S. Gogolla N. (2022). Harmonics of the social brain: how diverse brain regions coordinate appetitive social behavior. Neuron 110, 1608–1610. doi: 10.1016/j.neuron.2022.04.027, PMID: 35588713

[ref29] Lecca S. Meye F. J. Trusel M. Tchenio A. Harris J. Schwarz M. K.. (2017). Aversive stimuli drive hypothalamus-to-habenula excitation to promote escape behavior. eLife 6:e30697. doi: 10.7554/eLife.30697, PMID: 28871962 PMC5606847

[ref30] Li K. Zhou T. Liao L. Yang Z. Wong C. Henn F.. (2013). βCaMKII in lateral habenula mediates core symptoms of depression. Science 341, 1016–1020. doi: 10.1126/science.1240729, PMID: 23990563 PMC3932364

[ref31] Ma S. Chen M. Jiang Y. Xiang X. Wang S. Wu Z.. (2023). Sustained antidepressant effect of ketamine through NMDAR trapping in the LHb. Nature 622, 802–809. doi: 10.1038/s41586-023-06624-1, PMID: 37853123 PMC10600008

[ref32] Mathis V. Barbelivien A. Majchrzak M. Mathis C. Cassel J.-C. Lecourtier L. (2017). The lateral habenula as a relay of cortical information to process working memory. Cereb. Cortex 27, 5485–5495. doi: 10.1093/cercor/bhw316, PMID: 28334072

[ref33] Mathis V. Cosquer B. Avallone M. Cassel J.-C. Lecourtier L. (2015). Excitatory transmission to the lateral habenula is critical for encoding and retrieval of spatial memory. Neuropsychopharmacology 40, 2843–2851. doi: 10.1038/npp.2015.140, PMID: 25971591 PMC4864662

[ref34] Mathis V. Lecourtier L. (2017). Role of the lateral habenula in memory through online processing of information. Pharmacol. Biochem. Behav. 162, 69–78. doi: 10.1016/j.pbb.2017.07.004, PMID: 28709783

[ref35] Matsumoto M. Hikosaka O. (2007). Lateral habenula as a source of negative reward signals in dopamine neurons. Nature 447, 1111–1115. doi: 10.1038/nature05860, PMID: 17522629

[ref36] Matsumoto M. Hikosaka O. (2009). Representation of negative motivational value in the primate lateral habenula. Nat. Neurosci. 12, 77–84. doi: 10.1038/nn.2233, PMID: 19043410 PMC2737828

[ref37] Mendelsohn D. Riedel W. J. Sambeth A. (2009). Effects of acute tryptophan depletion on memory, attention and executive functions: a systematic review. Neurosci. Biobehav. Rev. 33, 926–952. doi: 10.1016/j.neubiorev.2009.03.006, PMID: 19428501

[ref38] Metzger M. Bueno D. Lima L. B. (2017). The lateral habenula and the serotonergic system. Pharmacol. Biochem. Behav. 162, 22–28. doi: 10.1016/j.pbb.2017.05.007, PMID: 28528079

[ref39] Moss E. H. Tantry E. K. Le E. Chin P.-S. Ambrosi P. Brandel-Ankrapp K. L.. (2025). Distinct patterns of PV and SST GABAergic neuronal activity in the basal forebrain during olfactory-guided behavior in mice. J. Neurosci. 45:e0200242025. doi: 10.1523/JNEUROSCI.0200-24.2025, PMID: 39965928 PMC11949486

[ref40] Nader K. Schafe G. E. Le Doux J. E. (2000). Fear memories require protein synthesis in the amygdala for reconsolidation after retrieval. Nature 406, 722–726. doi: 10.1038/35021052, PMID: 10963596

[ref41] Rock P. L. Roiser J. P. Riedel W. J. Blackwell A. D. (2014). Cognitive impairment in depression: a systematic review and meta-analysis. Psychol. Med. 44, 2029–2040. doi: 10.1017/S0033291713002535, PMID: 24168753

[ref42] Seyedabadi M. Fakhfouri G. Ramezani V. Mehr S. E. Rahimian R. (2014). The role of serotonin in memory: interactions with neurotransmitters and downstream signaling. Exp. Brain Res. 232, 723–738. doi: 10.1007/s00221-013-3818-4, PMID: 24430027

[ref43] Shumake J. Ilango A. Scheich H. Wetzel W. Ohl F. W. (2010). Differential neuromodulation of acquisition and retrieval of avoidance learning by the lateral habenula and ventral tegmental area. J. Neurosci. 30, 5876–5883. doi: 10.1523/JNEUROSCI.3604-09.2010, PMID: 20427648 PMC6632612

[ref44] Srivastava S. Arenkiel B. R. Salas R. (2023). Habenular molecular targets for depression, impulsivity, and addiction. Expert Opin. Ther. Targets 27, 757–761. doi: 10.1080/14728222.2023.2257390, PMID: 37705488 PMC10591939

[ref45] Stamatakis A. M. Stuber G. D. (2012). Activation of lateral habenula inputs to the ventral midbrain promotes behavioral avoidance. Nat. Neurosci. 15, 1105–1107. doi: 10.1038/nn.3145, PMID: 22729176 PMC3411914

[ref46] Swanson J. L. Ortiz-Guzman J. Srivastava S. Chin P.-S. Dooling S. W. Hanson Moss E.. (2022). Activation of basal forebrain-to-lateral habenula circuitry drives reflexive aversion and suppresses feeding behavior. Sci. Rep. 12:22044. doi: 10.1038/s41598-022-26306-8, PMID: 36543829 PMC9772215

[ref47] Tomaiuolo M. Gonzalez C. Medina J. H. Piriz J. (2014). Lateral Habenula determines long-term storage of aversive memories. Front. Behav. Neurosci. 8:170. doi: 10.3389/fnbeh.2014.00170, PMID: 24860453 PMC4026688

[ref48] Tronel S. Sara S. J. (2002). Mapping of olfactory memory circuits: region-specific c-fos activation after odor-reward associative learning or after its retrieval. Learn. Mem. 9, 105–111. doi: 10.1101/lm.47802, PMID: 12074998 PMC182591

[ref49] Vong L. Ye C. Yang Z. Choi B. Chua S. Lowell B. B. (2011). Leptin action on GABAergic neurons prevents obesity and reduces inhibitory tone to POMC neurons. Neuron 71, 142–154. doi: 10.1016/j.neuron.2011.05.028, PMID: 21745644 PMC3134797

[ref50] Xin J. Shan W. Li J. Yu H. Zuo Z. (2022). Activation of the lateral Habenula-ventral tegmental area neural circuit contributes to postoperative cognitive dysfunction in mice. Adv. Sci. (Weinh) 9:e2202228. doi: 10.1002/advs.202202228, PMID: 35616407 PMC9353455

[ref51] Xu Y. Lu Y. Cassidy R. M. Mangieri L. R. Zhu C. Huang X.. (2019). Identification of a neurocircuit underlying regulation of feeding by stress-related emotional responses. Nat. Commun. 10:3446. doi: 10.1038/s41467-019-11399-z, PMID: 31371721 PMC6671997

[ref52] Yang Y. Cui Y. Sang K. Dong Y. Ni Z. Ma S.. (2018a). Ketamine blocks bursting in the lateral habenula to rapidly relieve depression. Nature 554, 317–322. doi: 10.1038/nature25509, PMID: 29446381

[ref53] Yang Y. Wang H. Hu J. Hu H. (2018b). Lateral habenula in the pathophysiology of depression. Curr. Opin. Neurobiol. 48, 90–96. doi: 10.1016/j.conb.2017.10.024, PMID: 29175713

[ref54] Yang H. Yang J. Xi W. Hao S. Luo B. He X.. (2016). Laterodorsal tegmentum interneuron subtypes oppositely regulate olfactory cue-induced innate fear. Nat. Neurosci. 19, 283–289. doi: 10.1038/nn.4208, PMID: 26727549

[ref55] Zanos P. Gould T. D. (2018). Mechanisms of ketamine action as an antidepressant. Mol. Psychiatry 23, 801–811. doi: 10.1038/mp.2017.255, PMID: 29532791 PMC5999402

[ref56] Zarate C. A. Singh J. B. Carlson P. J. Brutsche N. E. Ameli R. Luckenbaugh D. A.. (2006). A randomized trial of an N-methyl-D-aspartate antagonist in treatment-resistant major depression. Arch. Gen. Psychiatry 63, 856–864. doi: 10.1001/archpsyc.63.8.856, PMID: 16894061

[ref57] Zhou W. Jin Y. Meng Q. Zhu X. Bai T. Tian Y.. (2019). A neural circuit for comorbid depressive symptoms in chronic pain. Nat. Neurosci. 22, 1649–1658. doi: 10.1038/s41593-019-0468-2, PMID: 31451801

