# Supplementary figures and images for: Glutamatergic lateral habenula neurons modulate consolidation of associative memories
Source: Front Behav Neurosci. 2025 Jul 29;19:1646689. doi: 10.3389/fnbeh.2025.1646689 (PMC12339511; doi:10.3389/fnbeh.2025.1646689)

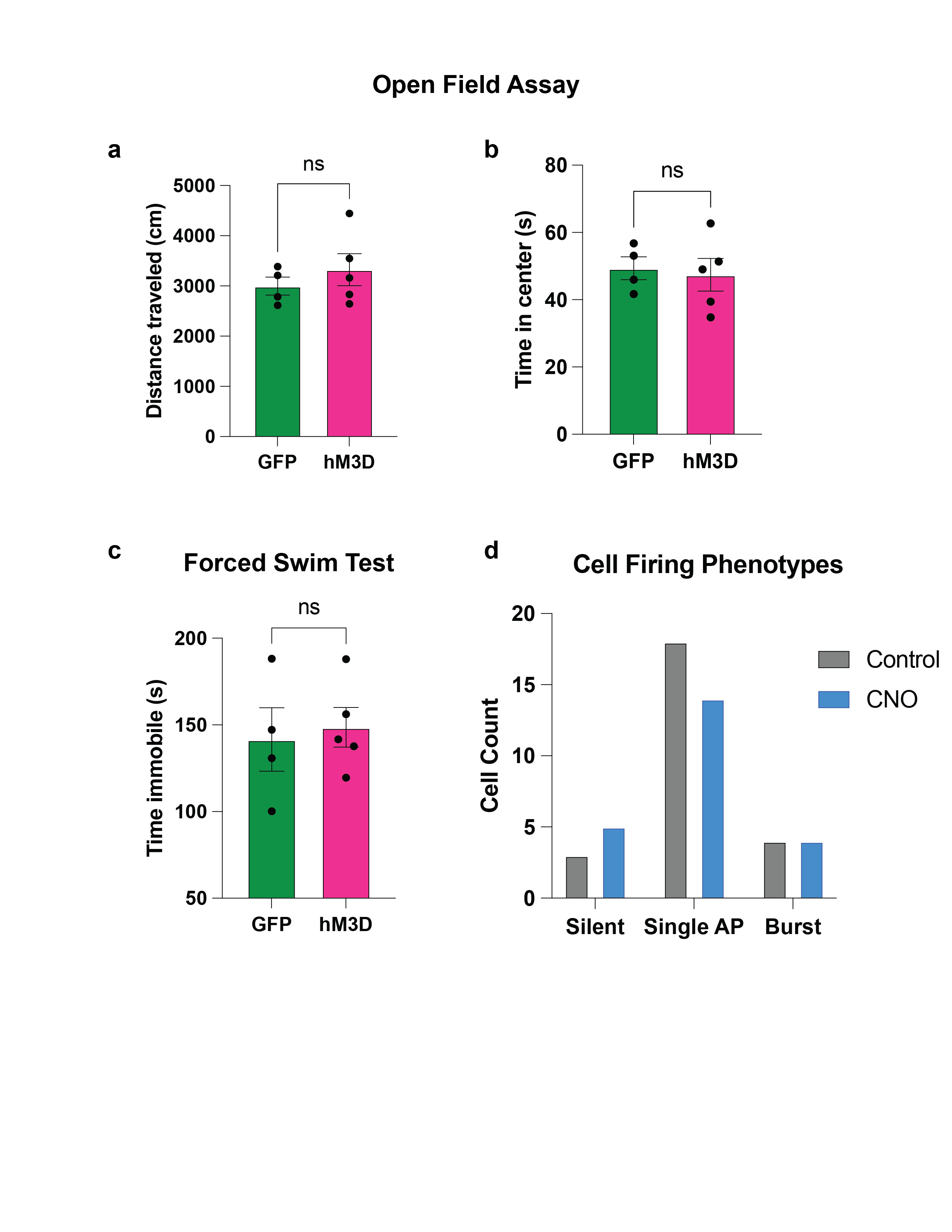

Supplement: SUPPLEMENTARY FIGURE 1 — hM3D activation of vGlut2+ LHb neurons does not drive anxiety or depression-associated phenotypes. (a) Differences in distance traveled during 10-minute open field exploration after CNO administration in hM3D and GFP control mice. Statistical significance calculated using Mann-Whitney test (p = 0.5556, alpha = 0.05). (b) Differences in time spent in center of arena during open field anxiety test between hM3D experimental (n = 5) and GFP control (n = 4) groups. Statistical significance calculated using Mann-Whitney test (p = 7302, alpha = 0.05). (c) Differences in time spent immobile during forced swim test depression assay between hM3D experimental (n = 5) and GFP control (n = 4) groups. Statistical significance calculated using Mann-Whitney (p = 0.9048, alpha = 0.05). (d) Number of vGlut2+ LHb cells exhibiting silent, single AP, and burst firing properties in ex vivo electrophysiological recordings of hM3D mice (n = 6) before and after bath application of CNO. Differences between control and CNO conditions evaluated using Chi-square test (Chi square = 0.918, df = 2, p = 0.632, alpha = 0.05). [file Supplementary_Figure_1.JPEG]
